# A systems science perspective on the capacity for change in public hospitals

**DOI:** 10.1186/s13584-017-0143-6

**Published:** 2017-03-24

**Authors:** J. Braithwaite, J. Westbrook, E. Coiera, W. B. Runciman, R. Day, K. Hillman, J. Herkes

**Affiliations:** 1 Centre for Healthcare Resilience and Implementation Science, Australian Institute of Health Innovation, Macquarie University, Sydney, NSW 2109 Australia; 2 Centre for Health Systems and Safety Research, Australian Institute of Health Innovation, Macquarie University, Sydney, Australia; 3 Centre for Health Informatics, Australian Institute of Health Innovation, Macquarie University, Sydney, Australia; 4 Centre for Population Health Research, School of Health Sciences, The University of South Australia, Adelaide, Australia; 5 St Vincent’s Clinical School, University of New South Wales, Sydney, Australia; 6 The Simpson Centre for Health Services Research, South Western Sydney Clinical School, The University of New South Wales, Sydney, Australia; 7 Intensive Care Unit, Liverpool Hospital, Sydney, Australia

**Keywords:** Health systems, Complexity science, Systems science, Organisational change, Public health

## Abstract

Many types of organisation are difficult to change, mainly due to structural, cultural and contextual barriers. Change in public hospitals is arguably even more problematic than in other types of hospitals, due to features such as structural dysfunctionalities and bureaucracy stemming from being publicly-run institutions. The main goals of this commentary are to bring into focus and highlight the “3 + 3 Decision Framework” proposed by Edwards and Saltman. This aims to help guide policymakers and managers implementing productive change in public hospitals. However, while change from the top is popular, there are powerful front-line clinicians, especially doctors, who can act to counterbalance top-down efforts. Front-line clinicians have cultural characteristics and power that allows them to influence or reject managerial decisions. Clinicians in various lower-level roles can also influence other clinicians to resist or ignore management requirements. The context is further complicated by multi-stakeholder agendas, differing goals, and accumulated inertia. The special status of clinicians, along with other system features of public hospitals, should be factored into efforts to realise major system improvements and progressive change.

## Background

It is a major challenge to attempt to introduce new regulations, policies, procedures or change programs, or even to simply try to nudge front-line behaviours, in order to alter the oftentimes rigid cultures and structures of hospitals [1]. Any efforts to disturb such a multi-faceted, intricate system requires the proponents of change to factor in the complexities of that system. Even slightly perturbing behaviours and cultures may create unintended as well as intended consequences, chiefly because hospitals are complex adaptive systems (CASs).

The characteristics of this type of intricate ecosystem include multiple, relatively autonomous agents interacting dynamically over time, and behaviours and outcomes that are unpredictable and emergent. This includes various degrees of randomness, conflict, cooperation, deception, redundant behaviours and, occasionally, incoherence.

Believing that the world of organisations, including public hospitals, is readily amenable to change by fiat or imposed design represents linear thinking—that the act of prescribing change directly leads to change. Such beliefs cannot wish away or ignore features of CASs such as natural inertia [2], politics [3, 4], embedded cultural characteristics [5], and bounded sub-structures [6, 7]. Hospitals, in effect, are not machines but organisms; [6, 8] more like frogs than bicycles [9].

## To what extent do public hospitals have the capacity to change?

In a recent *IJHPR article entitled* “Re-Thinking Barriers to Organizational Change in Public Hospitals” [10], Edwards and Saltman identify structural and contextual reasons that they contend are why public hospitals, chief amongst other types of organisations and institutions, are particularly resistant to change. They recognise the inherent properties of all hospitals: they are political, multi-stakeholder, and multi-dimensional. Yet many architects of change apply simplistic “solutions”, typically structural alterations in the form of formal redesigns. Our view is that these are pseudo-change, often resulting in little more than superficial rejigging of boxes on organisation charts. Such activities come in a number of guises e.g., “we should restructure” [11], “let’s recast the formal reporting arrangements” [12] or “we are going to invest in becoming more efficient by applying Lean techniques” [13].

This phenomenon is not restricted to hospitals. Indeed, it has been estimated that 70% of top-down, “mandated” or “directed” change initiatives across all industries, not just healthcare, fail outright or fall short of what proponents had planned for their organisations [14]. According to Edwards and Saltman, public hospitals are even harder than this to change, because of inherent entrenched politics and widespread anxieties at levels not present in organisations of other types. Summarised, their model suggests that the genesis of resistance to change in hospitals is two-fold: structural and contextual, and to explore this, they introduce a “3 + 3 Decision Framework” (Table 1).

**Table 1:** The “3 + 3 Decision Framework”

The 3 + 3 Decision Framework: sources of resistance to change	
Structural resistance group	Contextual resistance group
1. The dysfunctional characteristics found in most organizations	1. The inherent complexity of delivering high quality, safe, and affordable modern inpatient care in a hospital setting
2. The particular dysfunctions of professional health sector organizations	2. A set of specific market failures in public hospitals, which limit the scope of the standard financial incentives and reform measures
3. The additional dysfunctional dimensions of politically managed organizations	3. The unique problem of generalized and localized anxiety, which accompanies the delivery of medical services, and which suffuses decision-making on the part of patients, medical staff, hospital management, and political actors alike

*Source:* Summarised from Edwards and Saltman [10]

In each of these resistance groups, while several elements might pertain to many classes of organisation, they apply to a considerable degree, and in some cases are exclusive to, public hospitals. Edwards and Saltman argue for example, that the politics, under the structural domain, and the specific market failures, under the contextual domain, represent formidable barriers to change, acting to inhibit or negate the capacity for progress and improvement in public hospitals. Accordingly, they suggest that the totality of these structural and contextual factors make it difficult for policymakers and managers to formulate change strategies, make decisions, implement them, and create lasting improvements. Under such conditions, prescribing change from on high has little chance of success—rarely approaching the vision that the architects of change anticipated.

Whether it truly is the case that public hospitals are genuinely unique is a matter for debate. Resistance to change and inertia are well understood properties of many different classes of organisation, and even if these attributes were unique to public hospitals, they may not necessarily be anything more than local manifestations of widespread, deeply-embedded organisational structures and ingrained practices. Indeed, foundational attributes such as the tendency to accrete complexity with time is probably sufficient to trigger inertia or resistance, no matter the specific attributes of any particular organisation [2].

Regardless of whether they would agree with this, Edwards and Saltman’s solution for public hospitals is for stakeholders to use their “3 + 3 Decision Framework” to assess the potential of their reform initiatives before formulating or embarking on a target change program. The Framework thus acts as a kind of pre-implementation screening checklist, promoting early-stage discussions, applied prior to considering a change journey.

While we laud Edwards and Saltman for their attempt to tackle this thorniest of problems, we note that the Framework is directed primarily at decision-makers in the upper echelons of the systems they seek to change. Policymakers in national Ministries, CEOs of regional health districts, or managers of public hospitals all surely have a role to play in change and improvement, but a systems science perspective would take a broader stance than restricting the focus to the top of the hierarchy. The senior policy and managerial stakeholders certainly have responsibilities to set the organisational agenda. They have a major say in allocation of resources, and are responsible for guiding organisational direction. But real change requires the involvement—even in many cases the approval—of powerful stakeholders with less obvious, but nevertheless pervasive influence [15]. Of these, the most powerful stakeholder group is the clinicians, particularly medical clinicians.

## How clinicians and their characteristics fit in

Clinicians have strong within-profession collegiality, considerable collective capacity to resist, and deep-seated cultural characteristics which confer on them power sufficient that in most cases they decide on whether or not to accept mooted change [16], and how to process it if they do accept it. They strongly influence not just acceptance or rejection, but the pace of change. In short, clinicians have considerable capacity to manoeuvre. At their discretion, they can be aligned with or against the currents of change stimulated by top management. In any case, they always exercise subtle, informal, often hidden but powerful influences over organisational initiatives.

Thus, clinicians, especially doctors, can make or break virtually any change project. This is an evocative reminder that complex human systems like public hospitals are not merely complicated hierarchies, but comprise multi-stakeholder groups who have diffuse agendas and who are active players in determining organisational progress.

A feature of public hospitals which drives home these points is recognised by Edwards and Saltman. Public hospitals do change in a myriad of ways. New medical techniques, models of care, clinical practices, diagnostic technologies, tests and treatments are taken up in a continuous stream of adoption activities. Yet these are not the province solely of the senior executive staff, and nor are they embraced by the front line in response to policy determinations, releases of new procedures, or as managerially-sanctioned change programs. Adoption in public hospitals is much more bottom-up than top-down, and is largely initiated and sanctioned by clinical groups, although it does, of course, often require senior-level approval or funding or support.

This power of the front-line can be illuminated in other ways. In complex systems, there are identifiable stakeholder types, most of whom come from the coal-face clinical groups and are scattered across various low-level layers of the organisation who have a say, and exercise control over change, progress, and pace of adoption. They go by many names, including opinion leaders, mavens, bridges, liaisons, *tertius gaudens*, *tertius iungens*, connectors, and cosmopolites [17]. As well as being a product of the culture, individuals in these roles shape, guide or nudge it, thereby determining, through their interactions, the properties of the system [16]. These individuals, too, influence levels of receptivity to change, and organisational progress over time. Their attitudes and behaviours shape the dynamics of the system, and the boundary conditions under which it operates, often in far reaching ways.

These properties and characteristics of the public hospital environment, and the front-line actors within the system can be much more important influences than decisions taken remotely in the upper levels of the organisational structure. They contribute to the systems science phenomenon known as emergence—the patterns, properties and social structures that arise out of local interactive behaviours—in subtle and dynamic ways. This means that change in public hospitals is never under the commission or authority of any one group, and things—events, practices, projects—are always unpredictable.

Further, introducing a new intervention in an already complex system is unlikely to succeed unless a concomitant complexity reduction strategy is in place, effectively creating the headroom for the innovation. Addressing complexity head on, and reducing complexity to improve the chance of successful change, is likely to be a powerful generic approach to organisational change [2].

## Conclusion

In essence, systems science teaches us that we cannot hope to get close to a 1:1 correspondence between articulated change, prescribed or stimulated from above, and on-the-ground realised change [18]. Change designed by policy and managerial proponents is always shaped by, and interpreted idiosyncratically by, different stakeholder groups. Consequently, as useful as Edwards and Saltman’s model is, we would argue that we also need to factor in bottom-up characteristics, and to deeply appreciate the layered, socially textured nature of the CAS [4, 19, 20], for any meaningful, lasting improvements in public hospitals.

We can learn at least as much from the way clinicians actually handle, embrace or promote change at the coal-face, as we can from what regulators, policymakers and managers do when they mandate change. Perhaps future users of the “3 + 3 Decision Framework” could come to appreciate more these coal-face characteristics of the public hospital CAS before they embark on their next change initiative.
